# TCM targets ferroptosis: potential treatments for cancer

**DOI:** 10.3389/fphar.2024.1360030

**Published:** 2024-04-22

**Authors:** Liwen Qin, Yuhan Zhong, Yi Li, Yongfeng Yang

**Affiliations:** ^1^ Core Facilities of West China Hospital, Sichuan University, Chengdu, China; ^2^ Laboratory of Liver Transplantation, Frontiers Science Center for Disease-Related Molecular Network, West China Hospital, Sichuan University, Chengdu, China; ^3^ Department of Respiratory and Critical Care Medicine, Institute of Respiratory Health, Center of Precision Medicine, West China Hospital, Sichuan University, Chengdu, China

**Keywords:** ferroptosis, traditional Chinese medicine, cancer treatment, the role of ferroptosis, cancer

## Abstract

Ferroptosis is caused by the accumulation of cellular reactive oxygen species that exceed the antioxidant load that glutathione (GSH) and phospholipid hydroperoxidases with GSH-based substrates can carry When the antioxidant capacity of cells is reduced, lipid reactive oxygen species accumulate, which can cause oxidative death. Ferroptosis, an iron-dependent regulatory necrosis pathway, has emerged as a new modality of cell death that is strongly associated with cancer. Surgery, chemotherapy and radiotherapy are the main methods of cancer treatment. However, resistance to these mainstream anticancer drugs and strong toxic side effects have forced the development of alternative treatments with high efficiency and low toxicity. In recent years, an increasing number of studies have shown that traditional Chinese medicines (TCMs), especially herbs or herbal extracts, can inhibit tumor cell growth and metastasis by inducing ferroptosis, suggesting that they could be promising agents for cancer treatment. This article reviews the current research progress on the antitumor effects of TCMs through the induction of ferroptosis. The aim of these studies was to elucidate the potential mechanisms of targeting ferroptosis in cancer, and the findings could lead to new directions and reference values for developing better cancer treatment strategies.

## 1 Introduction

In the last decade, the Nomenclature Committee on Cell Death (NCCD) has meticulously examined an extensive body of literature, ultimately formulating a definition for cell death that incorporates considerations of morphology, biochemistry, and function. This definition remains applicable to the present day. In terms of functional characteristics, cell death modalities can be categorized into two types: accidental cell death (ACD) and regulated cell death (RCD). (Galluzzi et al., 2018). Ferroptosis, a novel type of regulated cell death, is morphologically, biochemically, and genetically distinct from apoptosis, necrosis, and pyroptosis, among others. Elevated levels of iron-dependent reactive oxygen species (ROS) result in the buildup of lipid peroxides, disrupting redox homeostasis and causing oxidative damage to cell membranes. This imbalance adversely impacts normal cellular processes, ultimately leading to ferroptosis (Xing et al., 2023).

In 2003, Dolma et al. reported that a new compound, erastin, could induce RAS-mutated human foreskin fibroblasts (BJeLRs) death. However, this pattern of cell death differs from classical apoptosis previously found. During erastin-induced cell death, there were observations of distorted mitochondrial morphology and the disappearance of mitochondrial cristae. (Dolma et al., 2003). However, no classic features of apoptosis, such as mitochondrial cytochrome c release, caspase activation, or chromatin fragmentation, are observed in RSL-treated cells (Yagoda et al., 2007; Yang and Stockwell, 2008). In Nine years later, this new mode of cell death was officially named ferroptosis by Dixon et al. (Dixon et al., 2012). Since then, researchers have continued to explore the relationship between ferroptosis and human disease. Although the specific mechanisms and physiological functions of ferroptosis have not been fully elucidated, increasing evidence indicates that ferroptosis is involved in multiple pathological conditions, such as ischemia‒reperfusion injury (Carlson et al., 2016), stroke mitigation and neurodegeneration (Hambright et al., 2017; Tuo et al., 2017), and cancer treatment (Conrad et al., 2016; Stockwell et al., 2017; Tonnus and Linkermann, 2017; Toyokuni et al., 2017). Several studies have shown that suppressing system xc- or GPX4 inhibits tumor growth and metastasis in various types of cancer (Yang et al., 2014; Zhang et al., 2019a). In addition, multiple tumor suppressors have been found to sensitize cells to ferroptosis. For example, p53 can enhance tumor ferroptosis by suppressing the transcription of the system xc-subunit SLC7A11 (Wang et al., 2016). Malignant mutations in oncogenes usually accelerate metastasis, protect cancer cells from apoptosis and increase resistance to common cancer therapies (Piccolo et al., 2014; Hansen et al., 2015). However, the finding that these same mutations sensitize cancer cells to ferroptosis brings new hope for cancer treatment (Wu et al., 2019).

Traditional Chinese medicine (TCM), a medical theory system gradually formed and developed through long-term medical practice under the guidance of ancient simple materialism and spontaneous dialectics, is one of the oldest medical systems in the world. It has condensed thousands of years of health-preserving concepts and practical experience. TCM and its natural extracts have been widely used to treat various diseases, making significant contributions to medical health worldwide. Traditional Chinese medicine has shown efficacy in treating acute pancreatitis (Ge et al., 2023), neurodegenerative diseases (Lin et al., 2023), and polycystic ovary syndrome (PCOS) (Liu et al., 2022). Myocardial infarction (MI) (Wang et al., 2023a), atherosclerosis (AS) and hyperlipidemia (HLP) (Chen et al., 2021a; Wu et al., 2023)develop through the activation of ferroptosis. Given the recent studies highlighting the association between ferroptosis and the inhibition of tumor cells, there is a growing suggestion that ferroptosis could be a potential target for anticancer therapy. (Chen et al., 2021b). Consequently, it is a novel and meaningful research direction to explore the ferroptosis induced by traditional Chinese medicine.

In this review, we list the traditional Chinese medicines and their natural extracts and prescriptions that can be used to treat different cancers. The mechanism of the ferroptosis pathway was investigated to guide rational clinical application, improve disease prognosis and reduce patient suffering caused by toxic side effects.

## 2 Main TCMs as ferroptosis regulators in cancer

### 2.1 Terpenoids

#### 2.1.1 Artemisinin and its derivatives

400 years ago, the famous Chinese herbalist Li Shi Zhen published in the Compendium of Materia Medica that “fever and colds” could be treated with qinghaosu preparations (Klayman, 1985). Artemisinin is indeed a natural sesquiterpene lactone compound. It is derived from the sweet wormwood plant (Artemisia annua). Since it was first isolated from the Asteraceae plant *Artemisia annua* by the Nobel laureate in Medicine and the Chinese scientist Tu Youyou in 1971, artemisinin has saved countless lives as an antimalarial drug (Bhattacharjee et al., 2018). Recently, the artemisinin and its derivatives not only save lives as antimalarials, but have also been explored as potential anticancer drugs.

Dihydroartemisinin (DHA), a derivative of artemisinin, can not only inhibit cell proliferation by inducing autophagy (Zou et al., 2019), but also exert its role by inhibiting the PRIM2/SLC7A11 axis and inducing ferroptosis (Yuan et al., 2020). The peroxide bridge structure of DHA triggers a Fenton reaction with the release of iron ions that may contribute to ferroptosis in tumor cells by promoting transferrin receptor expression and inhibits glutathione peroxidase (GPX4) (Greenshields et al., 2017; Yang et al., 2019; Su et al., 2021). DHA can induce ferritin lysosomal degradation and increases cellular free iron levels, increasing cellular susceptibility to ferroptosis. Furthermore, by binding to cellular free iron, thereby stimulating iron-regulatory proteins (IRPs) binding to mRNA molecules containing iron-responsive element (IRE) sequences. After the iron homeostasis controlled by IRP/IRE was broken by DHA, the cellular free iron continued to increase. Ferroptosis was induced in cancer cells by induction of GPX4 knockout *in vitro* and mouse xenograft models. DHA significantly increased the sensitivity of these cells to RSL3-induced ferroptosis and lipid peroxidation, including the human lung cancer cell line H292; the human colorectal cancer cell lines SW480, HCT116 and HT29; and the human breast cancer cell lines MDA453 and MCF-7 (Chen et al., 2020b).

Artesunate (ART) has exhibited antitumor effects on several nonurologic tumors. In both sunitinib-sensitive and sunitinib-resistant Renal Cell Carcinoma (RCC) cells, ART has been shown to inhibit proliferation and metastasis, and reduce metabolism. Artemisinin (ATS) substantially elevated cytotoxicity and suppressed proliferation in sunitinib-resistant RCC cells. In Caki-1, 786-O, and A-498 cell lines, the inhibition of growth was related to G0/G1 phase arrest and differential regulation of cell cycle-regulating proteins. Artesunate (ART) primarily impacts KTCTL-26 cells through ROS accumulation, ferroptosis, and decreased metabolism, as reported by Markowitsch et al., in 2020 (Markowitsch et al., 2020). Additionally, Roh et al. found that Artesunate (ART) selectively induces ferroptosis in head and neck cancer (HNC) cells while sparing normal tissue cells. Similarly, ART selectively killed cisplatin-resistant HNC cells without harming normal cells. As a pivotal transcription factor that regulates antioxidant stress, Nrf2 plays a crucial role in initiating the body’s antioxidant response. It controls the intricate cellular antioxidant system responsible for generating glutathione (GSH) in cancer cells. Artesunate (ART) has the ability to stimulate the generation of reactive oxygen species (ROS), which can result in ferroptosis, as well as other modes of cell death (Roh et al., 2017). ART can also suppress the proliferation of CA-46 cells *in vivo* through ferroptosis (Wang et al., 2019b). At the very least, ART has been shown to play a key role in improving the effectiveness of cancer treatment by inducing ferroptosis. It can also be combined with other antioxidants to enhance anticancer effects.

#### 2.1.2 Cucurbitacin B

Cucurbitacins, classified as tetracyclic triterpenoid natural products, are primarily derived from plants within the Cucurbitaceae family. These compounds showcase diverse pharmacological activities, including anti-inflammatory, hepatoprotective, antibacterial, antipyretic, and antitumor properties, achieved through the modulation of multiple signal pathways. Among these, Cucurbitacin B, extracted from Trichosanthes kirilowii Maximowicz, stands out as one of the most abundant and extensively researched derivatives of the cucurbitacin family. In traditional medicine, (Chen et al., 2005; Zhang et al., 2009; Chan et al., 2010a; Chan et al., 2010b; Dakeng et al., 2012). CuB could induce intracellular accumulation of iron ions and depletion of glutathione. A study revealed that CuB treatment of nasopharyngeal cancer cells downregulated GPX4 expression with iron accumulation and glutathione depletion, initiating extensive lipid peroxidation and ultimately leading to ferroptosis. Though, CuB significantly inhibited tumor progression and caused no significant side effects *in vivo* (Huang et al., 2021).

### 2.2 Phenols

#### 2.2.1 Curcumin

Curcumin, extracted from Curcuma longa L., has antioxidant properties and is now commonly used as a food additive. (Gao et al., 2022). A large number of studies have shown that curcumin inhibits the progression of non-small cell lung cancer (NSCLC) (Tang et al., 2021a), colorectal cancer (Miyazaki et al., 2023), follicular thyroid cancer (Chen et al., 2023b), clear cell renal cell carcinoma (ccRCC) (Xu et al., 2021a), and breast cancer (Li et al., 2020; Cao et al., 2022) by inducing ferroptosis. EF24, a synthetic analog of curcumin, induces ferroptosis through upregulating HMOX1 in osteosarcoma cells (Lin et al., 2021a). While Cao et al.proved that curcumin exerts anti-breast cancer activity by upregulating SLAC1A5 to induce lipid peroxidation and MDA accumulation (Cao et al., 2022).

Curdione, the predominant sesquiterpene in Curcumae Rhizoma (Xia et al., 2012), enhanced the expression of METTL14, a methylation transferase, and YTHDF2, a reader protein associated with m6A, through N6-methyladenosine. This leads to enhanced methylation of SLC7A11 mRNA and HOXA13 mRNA. Consequently, the expression of HOXA13 is reduced, resulting in a decrease in SLC3A2 expression. This intricate molecular modulation activates ferroptosis in colorectal cancer (Wang et al., 2023b). System Xc-, cystine/glutamic acid inverse transporter, can take cystine, excrete glutamic acid, both for intracellular glutathione synthesis to provide raw materials. SLC7A11 and SLC3A2 as transporter proteins play a key role in ferroptosis activated by GSH depletion. Inhibition of SLC7A11 expression serves as a trigger for inducing ferroptosis. Notably, HOXA13, functioning as a transcription factor, promotes the transcription of SLC3A2 and contributes to the promotion, growth, and therapeutic resistance observed in various malignancies (Gu et al., 2009; Quagliata et al., 2018; Ma et al., 2021).

#### 2.2.2 Erianin

Dendrobium was first recorded in the earliest Chinese pharmaceutical classic Shennong Bencao Jing. Erianin is a bibenzyl natural product extracted from Dendrobium chrysotoxum that have been reported to inhibit growth of cancer cells nowaday (Su et al., 2017; Ouyang et al., 2018; Zhang et al., 2019b). Chen, P., et al. reported After treatment of lung cancer cells with erianin, mitochondrial matrix coagulation and enlarged cristae of cancer cells were observed under electron microscope. In addition, HO-1 and TRF expression increased significantly, whereas GPX4, CHAC2, SLC40A1, SLC7A11 and glutaminase expression decreased significantly. This is the first demonstration that lanolin induces ROS accumulation, lipid peroxidation and GSH depletion in lung cancer cells, ultimately triggering ferroptosis (Chen et al., 2020b). Shen et al. confirmed that erianin significantly hampered the proliferation, invasion in Human renal cancer stem cells (HuRCSCs) while inducing oxidative stress injury and iron ion accumulation. Erianin’s mechanism of inducing ferroptosis in renal cancer stem cells involves inhibiting the expression of GPX4 and promoting the N6-methyladenosine modification of ALOX12/P53 mRNA. Ultimately, this process contributes to slowing down the development of renal cancer (Shen et al., 2023).

#### 2.2.3 Gallic acid

The process of obtaining gallic acid from gallnuts by fermentation was described in Li Ting’s Introduction to Medicine (1575) of the Ming Dynasty. Gallic acid (3,4,5-trihydroxybenzoic acid), a polyhydroxyphenolic compound, is the earliest documented organic acid (Hsu et al., 2011) that has inflammatory, antioxidant, antiviral, antianxiety and antineoplastic effects (Hsu et al., 2011; Mori et al., 2020), especially in liver cancer (Sun et al., 2016). Xie, J., et al. reported that GA could downregulate the expression of amino acid transporter SLC7A11 and ferroptosis signaling protein GPX4 in hepatocellular carcinoma cells by blocking β-catenin transport from the nucleus to the cytoplasm, thus inducing HepG2 ferroptosis (Xie et al., 2023).

### 2.3 Quinones

#### 2.3.1 Salvia miltiorrhiza bunge

Salvia miltiorrhiza Bunge, belonging to the Dicotyledonaceae family, is a perennial upright herb commonly known as Sage. In traditional Chinese medicine (TCM), the dried root and rhizome of salvia are utilized. This medicinal herb is known for its properties in activating blood circulation, removing stasis, dredging channels, relieving pain, and eliminating carbuncles (Song et al., 2013). Guan et al. documented that tanshinone IIA, extracted from Salvia miltiorrhiza, has the capacity to inhibit the proliferation of gastric cancer cells. This inhibitory effect is achieved through the induction of p53 upregulation-mediated ferroptosis (Guan et al., 2020). Haiwei Ni et al. demonstrated that tanshinone IIA suppresses the stemness of gastric cancer cells by triggering the activation of ferroptosis (Ni et al., 2022a). Lin et al. reported that dihydroisotanshinone I (DT), one of the main effective components of Salvia miltiorrhiza Bunge, has many biological activities and can inhibit the growth of breast carcinoma cells such as MCF-7 cells and MDA-MB-231 cells. In addition, DT treatment also significantly inhibited tumor proliferation in xenograft nude mice models *in vivo* without side effects. DT anti-tumor mechanism is associated with ferroptosis induced by down-regulating GPX4 protein expression (Lin et al., 2019).

A parallel mechanism is observed in another Danshen extract, cryptotanshinone (CTS), which exhibits the dual capability of activating caspase-3 to promote apoptosis and triggering ferroptosis in lung cancer by inhibiting GPX4 activity (Cao et al., 2021).

### 2.4 Saponins

#### 2.4.1 Ginsenoside

Ginsenoside is a steroidal compound found only in plants of the genus Ginseng and has a wide range of biological activities, including immunoregulatory (Shin et al., 2020), anti-inflammatory (Wang et al., 2018; Im, 2020), and antioxidative (Li et al., 2009) activities; in addition to its antitumor effects (Zhu et al., 2021). Rh4 inhibits colorectal cancer (CRC) cell proliferation by activating ROS/p53 signaling pathway, up-regulating p53 expression activates autophagy, downregulates GPX4, SLC7A11, and induces ferroptosis (Wu et al., 2022).

#### 2.4.2 Timosaponin (Tim-AIII)

Timosaponin AIII (Tim-AIII), classified as a steroid saponin, stands out as the primary active ingredient derived from Anemarrhena asphodeloides Bunge. Timosaponin AIII also has strong anticancer effects on liver cancer (Wang et al., 2013; Nho et al., 2016) and breast cancer (Sy et al., 2008; King et al., 2009), especially lung cancer (Zhou et al., 2023a). Zhou et al. disclosed that Timosaponin AIII (Tim-AIII) binds to HSP90, forming a complex that subsequently targets the degradation of GPX4 and promotes the ubiquitination of GPX4. Furthermore, Tim-AIII induces the accumulation of reactive oxygen species (ROS) and iron ions, as well as the production of malondialdehyde (MDA) and depletion of glutathione (GSH). These combined effects ultimately lead to therapeutic outcomes by inhibiting cell growth and reducing tumor volume through the induction of ferroptosis in non-small cell lung cancer (NSCLC) cell lines (Zhou et al., 2023b).

#### 2.4.3 Ophiopogon in B (OP-B)

Ophiopogon in B (OP-B) is extracted from Radix Ophiopogon japonicus and has been reported to exert anticancer effects on different types of cancer, such as nasopharyngeal carcinoma (Dong et al., 2021), hepatocellular carcinoma (Yuan et al., 2022), lung cancer (Cheng et al., 2022). Previous findings have shown that OP-B has anticancer effects through the noniron ferroptosis pathway (Chen et al., 2016; Zhang et al., 2022). OP-B has been observed to significantly degrade glutathione peroxidase 4 (GPX4) and solute carrier family seven member 11 (SLC7A11) following treatment of gastric cancer cells *in vitro*. Morever, the tumor volume and weight of AGS decreased after OP-B administration *in vivo*. It suggested that OP-B may induce ferroptosis in gastric cancer cells by inhibiting GPX4/Xc^−^, inducing ROS accumulation and glutathione deficiency (Zhang et al., 2022).

#### 2.4.4 Saikosaponin a (SsA)

Radix Bupleuri (RB) is derived from the dried roots of Bupleurum chinense or Bupleurum scorzonerifolium Willd. These plants are commonly found in sandy grasslands and dune meadows.

**FIGURE 1:**
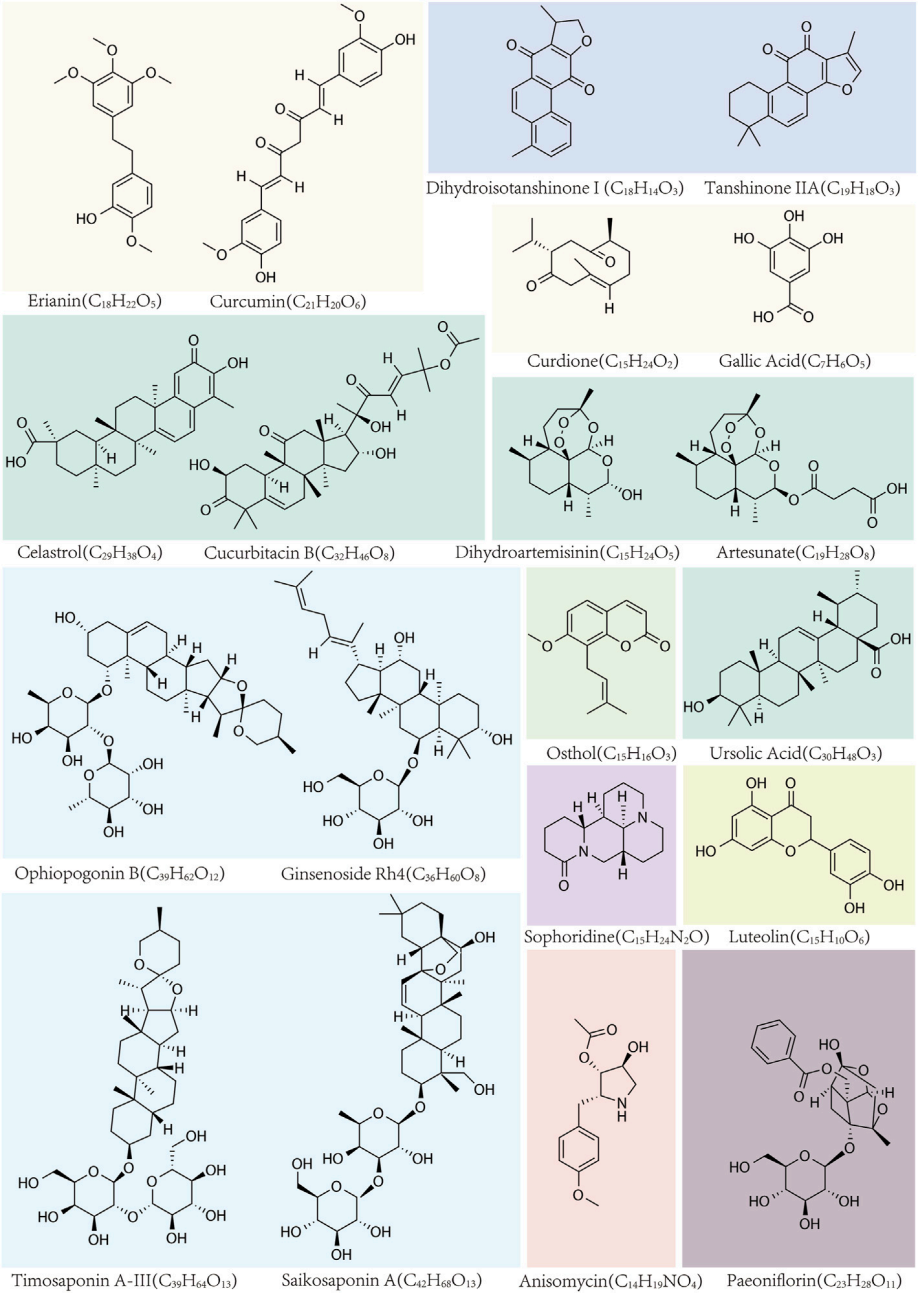
The names, structural formulas, and molecular formulas of TCMs that target ferroptosis for cancer treatment.

Saikosaponin A (SsA), a natural bioactive triterpenoid saponin extracted from RB (Wang et al., 2023a), has demonstrated potent antitumor activity against various types of tumors, including breast cancer (Zhang et al., 2021), cervical cancer (Du et al., 2021), pancreatic cancer (Shi et al., 2023), bladder cancer (Zhou et al., 2022), and colon cancer (Kang et al., 2017). Lan et al. reported that SsA induces ferroptosis in hepatocellular carcinoma (HCC) cells. This is achieved by activating endoplasmic reticulum (ER) stress-induced ATF3 upregulation and concurrently inhibiting the expression of the cystine transporter solute carrier family seven member 11 (SLC7A11) (Lan et al., 2023). (Figure 1)

### 2.5 Prescriptions

#### 2.5.1 Fu Fang Ku Shen injection (FKI)

Fu Fang Ku Shen injection has been widely used in adjuvant cancer therapy (Zhu et al., 2011; Zhao et al., 2014; Liu et al., 2017). Compared with single drugs, the combination of Fu Fang Ku Shen and chemotherapy drugs can improve leukaemia and relieve adverse reactions of digestive system gastric cancer (Yang et al., 2013; Lu et al., 2021a). Sophoridine is a natural plant monomer alkaloid obtained from Sophora alopecuroides and the main active compound of the Chinese traditional medicine Fu Fang Ku Shen (Qi et al., 2013). Sophoridine derivative 6j upregulates ATF3 expression through endoplasmic reticulum stress, promoting intracellular accumulation of iron ion, lipid oxygen species (ROS) and MDA, and activates ferroptosis in hepatocellular carcinoma cells (Tian et al., 2023).

#### 2.5.2 Qing Re Huo Xue Formula (QRHXF)

The main ingredient of Qing Re Huo Xue Formula are Radix Paeoniae Rubra and Scutellaria baicalensis (Xu et al., 2017). The majority of these chemical components play a role in regulating the biological process of oxidative stress and have an impact on the balance of antioxidants (Lin et al., 2021b; Han et al., 2022b). Studies have confirmed that Qing Re Huo Xue Fang has therapeutic effects on lung diseases including pulmonary fibrosis (Yang et al., 2023), chronic obstructive pulmonary disease (Lin et al., 2016) and lung cancer (Xu et al., 2017). Qing Re Huo Xue Formula significantly improved the accumulation of lipid ROS, iron ion and MDA while reducing GSH levels and strongly suppressed SLC7A11 and GPX4 protein levels. Qing Re Huo Xue Formula (QRHXF) activates ferroptosis to impede the progression of non-small cell lung cancer (NSCLC) cells through the involvement of the p53 signaling pathway (Xu et al., 2023).

### 2.6 Others

Red ginseng polysaccharide, an active ingredient of the herb Panax ginseng C. A. Meyer (Araliaceae), exhibits anticancer effects on human lung cancer and breast cancer. It achieves this by inducing ferroptosis, primarily through the targeting of GPX4 (Zhai et al., 2022).

Luteolin, a flavonoid, is naturally found in various medicinal herbs (Franza et al., 2021). It possesses a wide range of activities, including antioxidant (Ahmadi et al., 2020), anti-inflammatory (Wang et al., 2020a; Gendrisch et al., 2021), antifibrotic (Li et al., 2015), and anticancer properties (Prasher et al., 2022; Matić et al., 2023). Luteolin has been shown to induce ferroptosis in prostate cancer by promoting the nuclear translocation of transcription factor EB (TFEB) and enhancing ferritinophagy (Fu et al., 2023). Heme oxygenase-1 (HMOX1), an inducible enzyme, is considered a measurable indicator of oxidative stress (Maines, 1997). Previous reports have shown that targeting heme oxygenase-1 (HMOX1) can induce ferroptosis in liver cancer cells (Zheng et al., 2023) and ovarian cancer (Ni et al., 2023). Han et al. reported that luteolin exhibits an anticancer effect on clear renal cell carcinoma by upregulating heme oxygenase-1 (HO-1) expression, thereby triggering ferroptosis. It was demonstrated that luteolin exerted potent antitumor activity both *in vivo* and *in vitro*. (Han et al., 2022a). (Table 1)

**TABLE 1:** Active ingredients of TCMs targeting ferroptosis in cancer.

TCMs	Abbreviated name	Type	Effects	Cell	Cancer	Animal	References
Dihydroartemisinin	DHA	Terpenoids	PRIM2↓ SLC7A11↓β-Catenin↓	NCI-H23 XWLC-05	Lung cancer	Female nude mice xenograft tumor model	Yuan et al. (2020)
Dihydroartemisinin	DHA	Terpenoids	GPX4↓	NCI-H292	Lung cancer, Colorectal cancer	Athymic nude Foxn1nu/Foxn1+ mice xenograft tumor model	Chen et al. (2020a)
				HCT116, HT29, SW480, MDA-MB-453, MCF7	Breast cancer		
Dihydroartemisinin	DHA	Terpenoids	GPX4↓	MCF7/ADR	Breast cancer	-	Zhang et al. (2023)
Artesunate	ART	Terpenoids	Nrf2↓, HO-1↑, Keap1↑, p53↑	HN2–10	Head and neck cancer	Athymic BALB/c male nude mice (nu/nu) xenograft tumor model	Roh et al. (2017)
Artesunate	ART	Terpenoids	ER Stress (ATF4/CHOP/CHAC1 pathway)↑	DAUDI, CA-46	Burkitt’s lymphoma cell	NOD/SCID mice xenograft tumor model	Wang et al. (2019a)
Artesunate	ART	Terpenoids	GPX4↓	MCF-7, HeLa、 HepG 2, C6	Breast cancer, Liver cancer, *Rattus norvegicus* Glioma	Female athymic BALB/c-nude mice xenograft tumor model	Yu et al. (2023)
Cucurbitacin B	CuB	Terpenoids	GPX4↓, cyclinB1↓	MCF-7, A2780, CNE 1, HepG 2, H157, HCT-8	Breast carcinoma, Ovarian carcinoma, Nasopharyngeal carcinoma, Liver carcinoma, Lung carcinoma, Colorectal carcinoma	Female BALB/c nude xenograft tumor model	Huang et al. (2021)
Ursolic Acid	UA	Terpenoids	GPX4↓, TFR↑, NOCA4↓, Beclin-1↑	HOS, 134B	Osteosarcoma	NU/NU mice xenograft tumor model	Tang et al. (2021a)
Ursolic Acid	UA	Terpenoids	SLC7A11↓, Mcl-1	Hep3B, BEL-7402, H1299, A-427, SK-LU-1, T47D and MCF-7	Hepatoma, Lung cancer, Breast cancer	-	Li et al. (2022)
Curcumin	-	Phenols	SLC1A5↑, MDA↑	MDA-MB-453 and MCF-7	Breast cancer	Female BALB/c nude mice xenograft tumor model	Cao et al. (2022)
Curdione	-	Phenols	SLC7A11 mRNA N6-methyladenosine modification, METTL14↑, YTHDF2↑, GPX4 ↓, SLC7A11↓, HOXA13↓, SLC3A2↓	CT26, SW480	Colorectal cancer	BALB/c nude male mice subcutaneous transplantatio-n tumor model	Wang et al. (2023c)
β-elemene	-	Phenols	GPX4↓, SLC7A11↓, FTH1↓, SLC40A1↓, HO-1↑, Transferrin↑	HCT116, Lovo and Caco2	KRAS mutant colorectal cancer	Female BALB/c nude xenograft tumor model	Chen et al. (2020a)
Erianin	-	Phenols	ALOX12/P53 mRNA N6-methyladenosine Modification, GPX4↓, SLC7A11↓, GPX4↓, SLC7A11↓, FTH1↓, p53↑, ALOX12↑	HuRCSC	Renal cell carcinoma	BALB/Cnu/nu mice xenograft tumor model	Shen et al. (2023)
Erianin	-	Phenols	GPX4↓, SLC7A11↓, SLC40A1↓, Transferrin↑, CAM↑	H460, H1299	Lung cancer	Female BALB/c nude mice for the orthotopic xenograft lung tumor mouse model	Chen et al. (2020b)
Gallic Acid	GA	Phenols	GPX4 ↓, SLC7A11↓, Catenin↓	HepG2	Hepatocellular carcinoma	-	Xie et al. (2023)
DihydroisotanshinoneI	DT	Quinones	GPX4↓	MCF-7, MDA-23MB-231	Breast cancer	Male	Lin et al. (2019)
						BALB/c-nu and female nude mice	
Ginsenoside Rh4	-	Saponins	GPX4↓, p53↑, SLC7A11↓, SLC11A2↑, Nrf2↓, Beclin↑, LC3A/B↑, Atg7↑	HT29, HCT 116, DLD1, RKO	Colorectal Cancer	BALB/c nude mice xenograft tumor model	Wu et al. (2022)
Timosaponin AIII	Tim-AIII	Saponins	GPX4↓, HMOX1↑, SLC40A1↓, SLC7A11↓, FTL↓	H1299, A549	Non-small cell lung cancer (NSCLC)	C57BL/6J or BALB/c-nu/nu nude mice xenograft tumor model	Zhou et al. (2023a)
Ophiopogonin B	OP-B	Saponins	GPX4↓, xCT↓	NCI-N87, AGS	Gastric cancer	Female nude mice xenograft tumor model	Zhang et al. (2022)
Saikosaponin A	SsA	Saponins	ER stress, ATF3↑, GPX4↓, SLC7A11↓	HepG2, Huh-7	Hepatocellular carcinoma	Male BALB/c nude mice xenograft model	Lan et al. (2023)
Sophoridine Derivative 6j	6j		ER stress ATF3↑	HepG2, PLC/PRF/5, MHCC-97H, MHCC-97L, Bel-7402, K-Hep-1	liver cancer	Female BALB/C nude mice xenograft tumor model	Tian et al. (2023)
Anomanolide C	AC	Withanolide	GPX4↓	MDA-MB-231, BT-549	Triple negative breast cancer	FemaleBALB/c nude xenograft tumor model	Chen et al. (2023a)
Osthole	-	Coumarin	GPX4↓, SLC7A11↓, Transferrin↑ LAMP1↑ FTL↓ FTH↓ AMPKα^Thr172^↓ Akt^Ser473^↓ mTOR^Ser2448^↓	HCT 116 SW 480	Colorectal cancer	Female Balb/c nude mice xenograft model	Zhou et al. (2023a)
Paeoniflorin	PF	Glycoside	GPX4↓, NEDD4L↑, Nrf2↓	U251, U87	Glioma	Athymic nude mice subcutaneous xenograft tumor model	Nie et al. (2022)
Red ginseng polysaccharide	RGP	Polysaccharide	GPX4↓	A549, MDA-MB-231	Non-small cell lung cancer cell, triple-negative breast cancer cell	-	Zhai et al. (2022)
Luteolin	LUT	Flavonoids	GPX4↓, SLC7A11↓, LC3 I↓, LC3 II↑, TFEB↓, p62↓, Beclin↑	RWPE-1, DU145 PC-3, VCaP, LNcaP	Prostate cancer	Male nude mice BALB/c	Fu et al. (2023)
Luteolin	LUT	Flavonoids	HO-1↑, GPX4 ↓, SLC7A11 ↓, SLC40A1 ↓, FTL ↓, FTH1 ↓	786-O and OS-RC-2	clear cell renal cell carcinoma	Male BALB/c nude	Han et al. (2022a)
						mice xenograft tumor model	

## 3 TCMs in combination target ferroptosis

TCM can also be combined with other drugs to exert anticancer effects synergistically via ferroptosis (Table 3). DHA promotes lipid peroxidation and ROS accumulation through nanocarriers combined with tetrandrine (TET), and synergistically inhibits DOX-resistant breast cancer cell growth (Zhang et al., 2023). ART, another artemisinin derivative, in combination with Nrf2 inhibitors promotes ferroptosis in tumor cells, achieving a more potent anticancer effect without damaging normal cells. RSL3 or Artesunate alone in the mitochondria could inhibite GPX4 activity to trigger ferroptosis. While the mitochondria-targeting artemisinin/RSL3 nanomedicine, A/R-PLGA/CPT/DSSP, treatment resulted in the strongest GPX4 inhibition compared with RSL3-PLGA/CPT/DSSP or ART-PLGA/CPT/DSSP. Carbon centered free radicals and ROS are produced in mitochondria to induce ferroptosis (Yu et al., 2023). β-Elemene, derived and purified from the roots and stems of the traditional Chinese medicine turmeric, is classified as a second-class anticancer drug. It has been used to participate in the treatment of some cancers (Wang et al., 2012; Wang et al., 2019a; Xiaomeng et al., 2020; Chen et al., 2021a). β-Elemene as a complementary drug in combination with cetuximab inhibites tumor growth and migration of KRAS mutant CRC cells by inducing ferroptosis. (Chen et al., 2020c).

Ursolic acid (UA) is a naturally occurring triterpenoid that is widely found in common fruits and herbs. Previous studies have shown that UA can inhibit the proliferation of prostate, lung, pancreatic and other tumor cells by inducing apoptosis (Chen et al., 2019; Lin et al., 2020; Kornel et al., 2023). Ursolic acid not only synergized with low doses of cisplatin in a mouse osteosarcoma xenograft model, significantly reducing tumor growth, but also reduced cisplatin-induced weight loss in mice. Detailed molecular studies have shown that ursolic acid activates autophagy first, then degrades ferritin, intracellular ferrous ion overload, and triggers ferroptosis ultimately. In addition, ursolic acid elevated the ability of cisplatin to destroy DNA damage in osteosarcoma cells (Tang et al., 2021b). Notably, In another study, ursolic acid combined with sorafenib treatment of HCT116 resulted in accumulation of lipid ROS, and downregulation of the apoptosis-related proteins Mcl-1 and ferroptosis -associated protein SLC7A11. Therefore, these results suggest that the synergistic antitumor mechanism of sorafenib/UA may also trigger ferroptosis by inducing apoptosis. (Li et al., 2022).

Osthole, chemically known as 7-methoxy-8-(3-methyl-2-butenyl)-2H-1-benzopyran-2-one, is a natural coumarin extracted from Cnidium spp. and various other plants belonging to the Apiaceae family (Sun et al., 2021). An increasing number of studies have demonstrated that osthole has anticancer effects on a variety of cancers (Shokoohinia et al., 2018), including glioma (Huangfu et al., 2021), endometrial cancer (Liang et al., 2021), and colorectal cancer (Huang et al., 2014; Zhou et al., 2021). Zhou et al. revealed that osthole can reduce the phosphorylation of AMPK, Akt and mTOR in HCT116 and SW480 cells and induce Ferroptosis by inhibiting the AMPK/Akt/mTOR signaling pathway. Combinative treatment of β-elemene and cetuximab enhance anticancer effect of cetuximab. Thus osthole plays an antitumor role in colorectal cancer cells with KRAS mutations (Zhou et al., 2023a).

Sheng Mai Yin(SMY), as a decoction of traditional Chinese medicine, comes from the Jin Dynasty Chinese medicine classic “Medical Qiyuan,” which is mainly composed of Radix ginseng, Ophiopogon and Schisandra. According to the testing of researchers, it was found that the active ingredients of Sheng Mai Yin mainly include triterpenoid saponins, steroidal saponins, lignans, etc. (Zheng et al., 2009; Liu et al., 2016; Wang et al., 2020b; Xu et al., 2021b). Sheng Mai Yin has previously been reported to have therapeutic effects on acute lymphoblastic leukemia (Guo et al., 2023), cardiac hypertrophy (Ming et al., 2022), heart failure (Kan et al., 2022), type 2 diabetes mellitus (T2DM) (Li et al., 2019). In addition, Sheng Mai Yin has been shown to inhibit the toxic side effects of doxorubicin and induced ferroptosis by modulating HMOX1, assisting cancer treatment and reducing patient complications (Meng et al., 2023).

## 4 Summary and outlook

**FIGURE 2:**
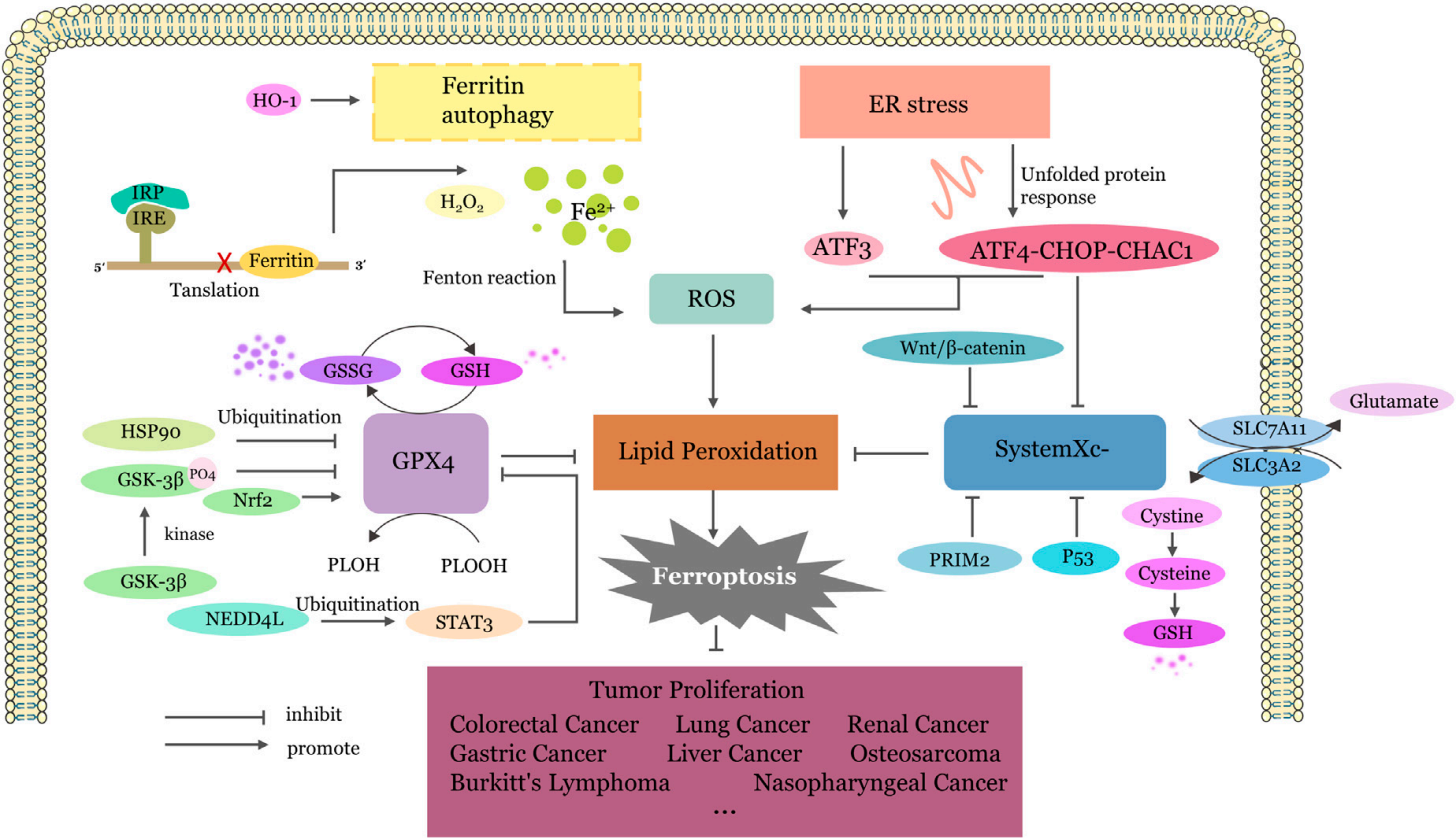
The Four Main Pathways of TCM Targeted Ferroptosis. Inhibiting GPX4:GPX4 undergoes ubiquitination degradation or translation inhibition mediated by different factors, thereby promoting the conversion of GSH to GSSG, reducing the reduction of phospholipid peroxides to phospholipid alcohols and increase lipid peroxidation. Inhibiting system Xc−: SLC7A11 and SLC3A2, as two components of the system Xc−, output glutamic acid and take up cysteine. Cysteine further participates in the synthesis of glutathione. When the system Xc− is inhibited, insufficient GSH conversion leads to the accumulation of lipid peroxidation products and ferroptosis. Activating endoplasmic reticulum stress: Upregulation of ATF3 expression or activation of the ATF4-CHOP-CHAC1 pathway by endoplasmic reticulum stress promotes the production of lipid ROS and inhibits system Xc−. Imbalance of iron ion homeostasis: Most Fe2+is stored in the form of an labile iron pool in ferritin. When ferritin autophagy occurs, excessive free iron is released, driving the Fenton reaction. The iron homeostasis regulation system IRP/IRE can be disrupted, receiving low iron signals, causing IRP to bind to the 5′ IRE, blocking ferritin synthesis, and promoting excessive free iron generation. ER, endoplasmic reticulum; ATF3, Activating Transcription Factor 3; ATF4, Activating Transcription Factor 4; GPX4, glutathione peroxidase 4; System Xc−,cystine/glutamate antiporter system; ROS, reactive oxygen species; CHAC1, Glutathione-specific gamma-glutamylcyclotransferase 1; PRIM2, DNA primase subunit 2; HSP90, Heat Shock Protein90; GSK3β, Glycogen synthase kinase 3β; GSH, Glutathione; GSSG, Glutathione disulfide; HO-1, heme oxygenase-1; IRP, Iron regulatory protein; IRE, iron-responsive element; HO-1, Heme oxygenase1; NEDD4L, Neural precursor cell expressed developmentally downregulated gene 4-like; STAT3, signal transducer and activator of transcription 3; Nrf2, nuclear factor erythroid 2-related factor 2; PLOH, phospholipid alcohol; PLOOH, phospholipid hydroperoxides; SLC7A11, Solute Carrier Family 7 Member 11; SLC3A2, Solute Carrier Family 3 Member two.

Since ferroptosis was first defined in 2012, it has become a hot topic in the field of various diseases. The relationship between traditional Chinese medicine and ferroptosis and the corresponding regulatory mechanism in cancer are new research directions. Although several articles have reported the anticancer effects of Traditional Chinese Medicines (TCMs) by regulating ferroptosis, there is a limited summary of the underlying mechanisms. Combining these research results, the anticancer effects of TCMs through ferroptosis can be broadly categorized into four pathways: (1) inhibiting GPX4, (2) inhibiting system Xc−, (3) activating endoplasmic reticulum stress, and (4) imbalance of iron ion homeostasis (Figure 2).

Glutathione peroxidase 4 (GPX4), an antioxidant enzyme belonging to the GPX protein family, is a critical aspect in defending against ferroptosis. GPX4, a selenium-containing cysteine enzyme, serves as a key defense mechanism against this form of cell death. Both GSH and GPX4 are important molecules in regulating cellular oxidative environment. GSH is also an essential cofactor of GPX4, so GPX4 is inhibited when GSH is depleted. (Cao and Dixon, 2016). It uses reduced GSH to convert toxic phospholipid hydroperoxides (PLOOHs) into nontoxic phospholipid alcohols (PLOHs) (Jiang et al., 2021). When GPX4 decreases, with an increase in toxic PLOOH, the membrane structure is destroyed to stimulate ferroptosis. (Stockwell et al., 2020). The Tim-AIII-HSP90 complex triggers ferroptosis in non-small cell lung cancer (NSCLC) through targeting ubiquitination and degradation of GPX4 (Zhou et al., 2023b). Anomanolide C (AC) reduces the expression of GPX4 through ubiquitination and inhibits triple-negative breast cancer (TNBC) proliferation and metastasis both *in vitro* and *in vivo* (Chen et al., 2023a). Paeoniflorin suppressd Nrl2 and GPX4 via upregulation of NEDD4L and ubiquitination of STAT3 (Nie et al., 2022). Dihydroisotanshinone I induces ferroptosis in Breast cancer cells by inhibiting GPX4, leading to depletion of intracellular glutathione and a sharp increase in GSSG(Ahmadi et al., 2020). Similarly,OP-B (Zhang et al., 2022) and cucurbitacin B (Huang et al., 2021)induce ferroptosis by inhibiting GPX4.

The cystine/glutamate reverse transport system (system xc -) exports glutamate out of the cell and imports cystine into the cell in equivalent proportions, which plays a key role in the synthesis of glutathione (GSH), an important antioxidant (Niu et al., 2021). Upon entering the cell, cystine undergoes rapid conversion to L-cysteine, playing a pivotal role as a key constituent in the synthesis of intracellular glutathione (GSH). Glutathione is a substance that contains γ- Tripeptides with amide bonds and thiol groups, as antioxidants, are present in almost every cell of the mammalian body. It serves to restore intracellular reduction‒oxidation (REDOX) balance subsequent to reactive oxygen species (ROS) production, thus preventing cellular damage from free radicals, peroxides, and lipid peroxides. Additionally, it acts as an essential substrate for the enzymatic activity of GPX4 (Niu et al., 2021). Inhibiting the Xc− system indirectly results in GSH depletion, disrupting endogenous antioxidant mechanisms and leading to a substantial accumulation of ROS, ultimately triggering ferroptosis (Yu et al., 2017). Iron overload, accumulation of reactive oxygen species (ROS) and phospholipid hydroperoxides (PLOOH), initiation of Fenton reaction and further release of PLOOH are markers of Ferroptosis (Conrad and Pratt, 2019; Liang et al., 2022). SLC7A11, an amino acid transporter, regulates cystine uptake and the biosynthesis of glutathione and promotes the establishment of the antioxidant defense system. Targeted inhibition of SLC7A11 promotes intracellular lipid peroxide accumulation, induce tumor cell ferroptosis, and enhances sensitivity to immunotherapy, radiotherapy, and chemotherapy (Koppula et al., 2021; Yan et al., 2023). Dihydroartemisinin downregulate the level of PRIM2/SLC7A11 axis so that induce ferroptosis and inhibits the proliferation of lung cancer cell (Yuan et al., 2020). Gallic acid inhibit SLC7A11 and Wnt/β-catenin signaling to promote hepatocellular carcinoma ferroptosis (Xie et al., 2023). Qingrehuoxue Formula activated GSK-3β phosphorylation while reduced GSH and SLC7A11 level. Ferroptosis in NSCLC is subsequently activated with the accumulation of ROS and MDA (Xu et al., 2023). Curcumin suppresses breast cancer by elevating solute carrier family one member 5 (SLC1A5) and enhanced glutamine uptake (Cao et al., 2022).

Endoplasmic reticulum (ER) stressis a cellular response triggered by protein misfolding, accumulation of unfolded proteins, and disturbances in calcium homeostasis within the ER lumen. This activates unfolded protein responses, ER overload responses, and, in severe cases, apoptosis (Wei et al., 2021). There is increasing evidence suggesting a close relationship between ferroptosis and endoplasmic reticulum (ER) stress (Dixon et al., 2014). Moreover, the mechanism by which certain natural products induce ferroptosis is closely related to endoplasmic reticulum (ER) stress (Lu et al., 2021b). Saikosaponin A (SsA) (Lan et al., 2023) and the sophoridine derivative 6j (Tian et al., 2023) induced ferroptosis in hepatocellular carcinoma (HCC) cells by activating ER stress. Artesunate (ART) enhances ferroptosis in Burkitt’s lymphoma cell lines by activating the ATF4/CHOP/CHAC1 pathway, an endoplasmic reticulum stress response (Wang et al., 2019b).

Some studies have suggested that ferroptosis is an autophagy-dependent form of cell death. Excessive autophagy can activate ferroptosis through the accumulation of iron ions or lipid reactive oxygen species (ROS). Ferritin autophagy, a process in which ferritin is degraded through autophagy, has been identified as triggering ferroptosis in various cancer cells (Gao et al., 2016; Hou et al., 2016). Luteolin (Fu et al., 2023), Rh4 (Wu et al., 2022) and ursolic acid (Tang et al., 2021a)promote ferroptosis by increasing autophagy. Ferritin is a cytoplasmic iron storage protein, negative feedback protein of Ferroptosis. It consists of two subunits, ferritin heavy chain 1 (FTH1) and ferritin light chain (FTL) (Xing et al., 2023). When ferritin autophagy degrades, it releases large amounts of free ferrous ions that combine with hydrogen peroxide to trigger the Fenton reaction that activates Ferroptosis (Dixon et al., 2012). Therefore, the death mode of ferritinophagy is essentially an iron metabolism disorder in cells, and iron homeostasis is unbalanced. Luteolin demonstrates an anticancer effect on clear renal cell carcinoma by upregulating heme oxygenase-1 (HO-1) expression and activating labile iron pool (LIP)directly, thus triggering ferroptosis (Han et al., 2022b).

**FIGURE 3:**
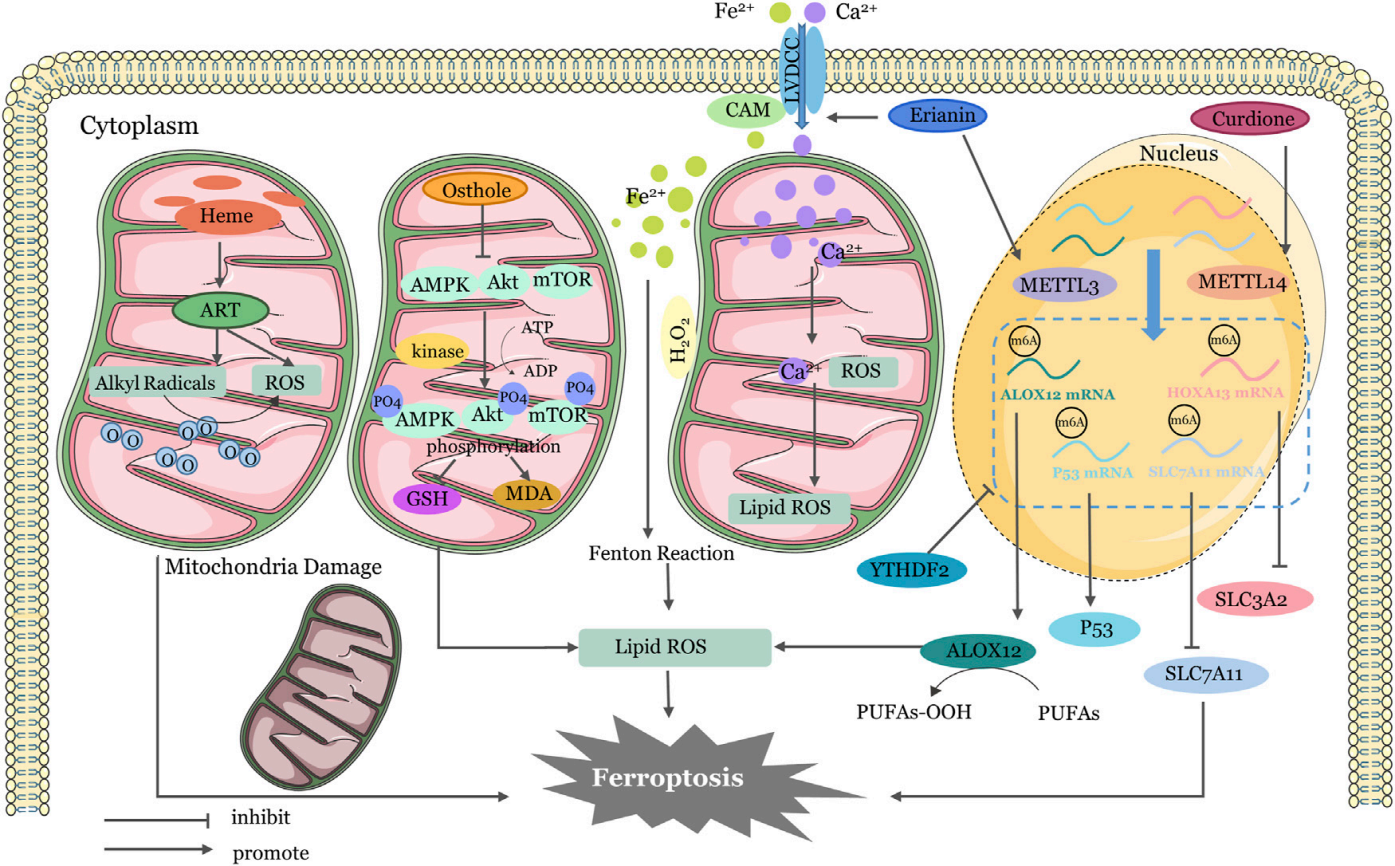
Mechanisms of Four Other Targeted Ferroptosis Pathways in TCMs. Activation of ART by heme produces ROS and alkyl radicals in cancer cell mitochondria, which damage membrane structures and damage mitochondria. Osthole supresses GSH generation and lipid ROS accumulation by activating AMPK/Akt/mTOR pathway phosphorylation. Erianin-activated calmodulin regulates LVDCC, increasing absorption of Fe2+ and Ca2+. Excessive calcium and iron ions induce lipid peroxidation. Erianin upregulates METTL3 to promote ALOX12 and P53mRNA methylation and increases ROS accumulation. Curdione upregulates METTL14 to promote SLC7A11mRNA and HOXA13mRNA methylation, reduce YTHDF2 stability, and inhibit expression of SLC7A11 and SLC3A2. ER, endoplasmic reticulum; ATF3, Activating Transcription Factor 3; ATF4, Activating Transcription Factor 4; GPX4, glutathione peroxidase 4; System Xc−,cystine/glutamate antiporter system; ROS, reactive oxygen species; CHAC1, Glutathione-specific gamma-glutamylcyclotransferase 1; PRIM2, DNA primase subunit 2; HSP90, Heat Shock Protein90; GSK3β, Glycogen synthase kinase 3β; GSH, Glutathione; GSSG, Glutathione disulfide; HO-1, heme oxygenase-1; IRP, Iron regulatory protein; IRE, iron-responsive element; HO-1, Heme oxygenase1; NEDD4L, Neural precursor cell expressed developmentally downregulated gene 4-like; STAT3, signal transducer and activator of transcription 3; Nrf2, nuclear factor erythroid 2-related factor 2; PLOH, phospholipid alcohol; PLOOH, phospholipid hydroperoxides; SLC7A11, Solute Carrier Family 7 Member 11; SLC3A2, Solute Carrier Family 3 Member two.

In addition to the four main pathways mentioned above earlier, there are other ways of regulation (Figure 3). Erianin has the capacity to induce ferroptosis in renal cancer stem cells by promoting the N6-methyladenosine modification of ALOX12/P53 mRNA (Shen et al., 2023). Curdione can activate ferroptosis by enhancing the methylation of SLC7A11 mRNA and HOXA13 mRNA (Wang et al., 2023b). Erianin triggered ferroptosis in lung cancer cells by activating Ca2+/CaM signaling (Chen et al., 2020c). Heme activates artemisinin to generate alkyl radicals and/or ROS in mitochondria of cancer cells. RSL-3 inhibits GPX4 and further induces mitochondrial lipid peroxidation (Yu et al., 2023). AMP-activated protein kinase (AMPK) has been shown to inhibit tumor growth by resisting ferroptosis in some studies (Lee et al., 2020). Yi et al. have suggested that upregulation of PI3K-AKT-mTOR signaling inhibit ferroptosis though SREBP-mediated lipogenesis (Yi et al., 2020). Activation of PI3K/AKT/Nrf2 was shown to improve cognitive impairment after cerebral ischemia by up-regulating GPX4 to inhibit ferroptosis (Fu et al., 2022). Similarly, osthole decreases AMPK phosphorylation and exactly promotes ferroptosis in KRAS-mutant colorectal cancer cells (Zhou et al., 2023a). These regulatory pathways involving key physiological and biochemical targets shed light on whether we might be able to use related factor inhibitors to aid in cancer prevention in the future. Certainly, this needs to be studied more thoroughly and in conjunction with clinical trials.

Indeed, several researchers have employed nanoparticles as tools to induce ferroptosis in tumor cells. The unique properties of nanoparticles make them promising agents for targeted therapeutic strategies, including the induction of ferroptosis in cancer cells (Jiang et al., 2021). For example, Ni et al. used atranorin complexes comprising superparamagnetic iron oxide nanoparticles (SPIONs) (Atranorin@SPION) to induce ferroptosis in GCSCs by decreasing the expression level of the Xc^−^/GPX4 axis and enhancing the 5-hydroxymethylcytosine modification of mRNAs in the pathway, thereby achieving therapeutic effects on gastric cancer (Ni et al., 2022b). Additionally, Yu et al. first demonstrated that artemisinin, a mitochondrial targeting agent, was much more toxic *in vitro* and *in vivo* than free artemisinin and non-targeted artemisinin nanodrugs against a variety of cancer cells, including MCF-7, HeLa, HepG2 and C6 cells. Mitochondrial artemisinin toxicity is mainly caused by free radicals and/or ROS-related apoptosis associated with artemisinin induced ferroptosis (Yu et al., 2023).

Some studies have also compared the effects of TCMs with classical ferroptosis inducers RSL-3 or erastin, which tend to increase the sensitivity of tumor cells to ferroptosis, resulting in mutual promotion (Table 2). Additionally, TCMs not only promote anticancer effects alone but also produce additive or synergistic effects when combined with Western medicines (Table 3). On the one hand, TCMs enhance the inhibition of tumor growth and metastasis by targeting iron death, and on the other hand, they alleviate the toxic side effects caused by chemotherapy drugs. After all, drug toxicity is an important consideration in terminal treatment options for cancer patients. These advantages make TCMs promising antitumor drugs.

**TABLE 2:** TCMs impact on classic inducers (erastin or RSL3) of ferroptosis.

TCMs	Cell	Effect	Mechanism	References
Dihydroartemisinin	MEFs,HT1080	Promotes cysteine starvation (STV)-induced ferroptosis in a time-and dose-dependent manner	Sensitizes cells to ferroptosis through inducing the lysosomal degradation of ferritin and IRE/IRP axis suppressing ferritin synthesis	Chen et al. (2020b)
		Improved the sen sitivity of erastin,RSL-3,FIN56		
		Increase lipid peroxide generation		
Artesunate	HN3-cisR, HN4-cisR, and HN9-cisR	Decreases cisplatin-resistant HNC cell lines glu-tathione (GSH) levels and causee lipid ROS accumulation	like the ferroptosis inducer erastin, activates Nrf2 by inhibiting Keap1	Roh et al. (2017)
Artesunate	MCF-7,COS7	Mitochondria-targeting ART and RSL3 nanomedicine synergistically enhances anticancer effect *in vitro* and *in vivo*	Heme activates ART to generate a lot of ROS in mitochondria, further enhancing GPX4 inhibitor RSL3 induced ferroptosis	Yu et al. (2023)
Paeoniflorin	U251, U87	Combination of RSL3 and PF more signifcantly inhibites cell proliferation	Inhibit Nrf2 and GPX4 by regulating NEDD4L	Nie et al. (2022)

**TABLE 3:** TCMs used in combination with other drugs.

TCMs	Drugs	Combination medication	Ferroptosis effect	Alleviate the toxic side effects	References
Dihydroartemisinin	Tetrandrine (TET)	DHA-TET pH-sensitive LPs	Exerts synergistic effects in tumor cell proliferation and inhibit doxorubicin (DOX) resistance	Reducing the cardio-toxicity from doxorubicin (DOX)	Zhang et al. (2023)
Artesunate	RSL-3	A/R-PLGA/CPT/DSSP nanomedicine (A/R = 11.1/1)	Exerts synergistic effects of anticancer *in vitro* and *in vivo* by activation of ART and generation of alkyl radicals and/or ROS in mitochondria	The cytotoxicity against nomal cells (COS 7) was significantly lower than that of cancer cells (MCF-70)	Yu et al. (2023)
β-elemene	Cetuximab	β-elemene (125 μg/mL) was combined with cetuximab (25 μg/mL) or intraperitoneally	Inhibites KRAS mutant tumor growth and suppresses the	No notable	Chen et al. (2020c)
		injected with 100 μL of PBS, β-elemene (50 mg/kg),	migration of KRAS mutant CRC cells	Toxicity was found in organs after H&E stained	
		cetuximab (50 mg/kg)			
Ursolic Acid	Cisplatin	35 μM UA combined with 20 μM Ci	Exerts synergistic effects with cisplatin on inhibiting tumour cell proliferation by activating autophagy and degrade ferritin	Reduced the toxicity and side effects of Cis	Tang et al. (2021b)
Osthole	Cetuximab	50 μM osthole combined with 100 ng/mL cetuximab or 20 mg/kg osthole combined with 10 mg/kg cetuximab	Increased the anticancer effect of cetuximab by reducing phosphorylation of AMPK/Akt	-	Zhou et al. (2023b)
Sheng-Mai-Yin	DOX	SMY-L (135 mg/kg/day) group, SMY-H (270 mg/kg/day) and receivrd DOX (10 mg/kg)	Reduced iron overload and lipid oxidation by inhibiting expression of HMOX1	Reducing the cardio-toxicity from doxorubicin (DOX)	Meng et al. (2023)
		intraperitoneally on the sixth day			

Despite rapid and promising advances in research into the role of TCMs in iron death in cancer therapy, implementation into clinical use remains challenging. Activating ferroptosis can kill cancer cells, but whether normal tissues are damaged at the same time raises concerns about potential complications associated with the use of iron death inducers (Sui et al., 2019). Moreover, there are still doubts about the nonstandard compatibility, inaccurate dosage and nonsingle extract of traditional Chinese medicine. While Traditional Chinese Medicines (TCMs) and their active ingredients have been reported to have regulatory effects on ferroptosis, encompassing additional targets, stable structures, high safety, low cost, and easy availability, there is a notable insufficiency in the accumulation of related studies. Clinical trials are also lacking, and for many drugs, the mechanisms of action have not been fully elucidated. Therefore, to apply TCMs more rigorously and scientifically in the clinical treatment of cancer, future research should include additional large sample multicenter double-blind randomized controlled trials and related molecular cell biology experiments to further examine the mechanism of action, effectiveness and safety of TCMs. We hope that eventually TCMs can really slow down the progression of the disease, alleviate the suffering of patients, and improve the quality of life.
